# Involvement of p38 MAPK in the Drug Resistance of Refractory Epilepsy Through the Regulation Multidrug Resistance-Associated Protein 1

**DOI:** 10.1007/s11064-015-1617-y

**Published:** 2015-06-20

**Authors:** Cuicui Wang, Zhen Hong, Yinghui Chen

**Affiliations:** Department of Neurology, Jinshan Hospital, Fudan University, 1508 Longhang Road, Shanghai, 201508 China; Department of Neurology, Huashan Hospital, Fudan University, 12 Mid Wulumuqi Road, Shanghai, 200040 China; Department of Neurology, Shanghai Medical College, Fudan University, Shanghai, 200032 China

**Keywords:** p38 MAPK, Refractory epilepsy, Drug resistance, Multidrug resistance-associated protein 1

## Abstract

Increased expression of multidrug-resistance associated protein 1 in brain tissue has been reported which lead to multidrug resistance of refractory epilepsy. However, the mechanism of up-regulated expression is still unclear. In our previous study, we have found that the MAPK signaling pathway mediated the expression of P-glycoprotein. So in this study, we used a rat model of refractory epilepsy to examine whether p38 MAPK affect the expression of MRP1 and the concentrations of AEDs in the brain. The expression of MRP1 and p38 MAPK was detected by immunofluorescence, Western-blot and real time-PCR, while the concentration of AEDs was measured by microdialysis and HPLC. The result showed that SB202190, the specific inhibitor of p38 MAPK, could down-regulate the expression of MRP1, while increase the concentrations of valproate and lamotrigine in hippocampus extracellular fluid of refractory epileptic rat. We demonstrate that p38 MAPK signaling pathway may be involved in drug resistance of refractory epilepsy by regulating MRP1.

## Introduction

Refractory epilepsy is a common neurologic disease but remains a major medical intractability that accounts for approximately one-third of epileptic patients with resistance to several, if not all, antiepileptic drugs (AEDs), even though these drugs act by different mechanisms [1, 2]. The therapeutic effect of treatment of epilepsy is limited by the development of multidrug resistance, which mainly induced by intrinsic or acquired overexpression of efflux pumps in the blood–brain barrier (BBB), such as P-glycoprotein and multidrug-resistance associated protein 1 (MRP1) [3]. Therefore, the search for down-regulation expression of multidrug resistance transporter is a rational approach to solve the drug resistance. p38 MAPK is a member of the MAPK family, which produce certain biological effects through the activation of some transcription factors and other proteases. There is now some evidences that inhibition of the p38 MAPK signaling pathway down-regulated P-gp expression and diminished the cellular multidrug resistance [4–6]. In addition, there was showed that p38 MAPK and ERK were involved in the resistance of cells to mercury by expression MRP1 in AML-2/DX100 cells [7]. The above suggest that p38 MAPK signaling pathway may be involved in the drug resistance of multidrug transporter mediated refractory epilepsy and plays an important role. In this study, the expression of MRP1 in the brain of refractory epilepsy rat was investigated, while the effect of the inhibition of p38 MAPK on the concentrations of two AEDs, VPA and LTG [8], in hippocampal extracellular fluid of epilepsy model was also evaluated. It was demonstrated that whether p38 MAPK is involved in drug resistance of refractory epilepsy through regulation the expression of MRP1.

## Materials and Methods

### Chemicals

Pentylenetetrazol (PTZ), phenytoin (PHT), sodium valproate (VPA), lamotrigine (LTG), Dapi, and SB202190 were purchased from Sigma-Aldrich Co. (St. Louis, MO, USA). Mouse monoclonal antibody against MRP1 was purchased from Abcam plc. (Cambridge Science Park, Cambridge, UK). Rabbit monoclonal antibody against p38 MAPK was obtained from Cell Signaling Technology Inc. (Danvers, MA, USA). Mouse monoclonal antibody against phosphorylate-p38 (p-p38), mouse polyclonal antibody against GAPDH, goat anti-mouse IgG-HRP and goat anti-rabbit IgG-HRP were obtained from Santa Cruz Biotechnology Inc. (Santa Cruz, CA, USA). DyLight™488-conjugated affinipure goat anti-mouse IgG and DyLight™594-conjugated affinipure goat anti-rabbit IgG were from Jackson Immuno Research laboratories, Inc. (West Grove, PA, USA). TRIzol™ reagent and a reverse transcription system were purchased from Promega (Madison, WI, USA). Sequences of oligonucleotide primers for real time-PCR analysis were synthesized by Bio TNT Inc. (Shanghai, China). PrimeScript™ RT master mix and PrimeScript™ RT reagent kit with gDNA Eraser were purchased from TaKaRa (Otsu Shiga, Japan). Microdialysis probe, microdialysis cannula and CMA/100 micro-injection pump came from CMA Inc. (Derwood, MD, USA). MonEL510 high performance liquid chromatography was made from Beijing Syltech Scientific Instrument Co. (Ltd, Beijing, China).

### Animals and Grouping

Male Wistar rats (weighing 200–250 g) were purchased from SIPPR/BK laboratory animal Ltd (Shanghai, China). All animal experiments were approved by the Guideline for Animal Experiments of the Chinese. The rats were housed in standard animal-grade room with one animal in each cage. The temperature was maintained at 20 ± 2 °C, the relative moisture at 60 %, and the light cycle at 12 h/day. The rats were allowed to adapt to the new conditions for at least 1 week before being used in the experiments.

Animals were randomly divided into three groups: control group, refractory epilepsy group (epilepsy group) and SB202190 group. The refractory epileptic rats were administrated with SB202190 by the microdialysis probe in the SB202190 group, while artificial cerebrospinal fluid was administrated by microdialysis in epilepsy group. Each group included eight rats.

### Establishing of a Epileptic Rat Model

Rats were intraperitoneally injected with 1 % PTZ at 40 mg/kg body weight every other day for 14–20 days [9]. After PTZ injection, their behavior was continuously observed for a period of 30 min. Seizure severity was evaluated according to the standards of Racine [10]. Rats with three consecutive stage V seizures were considered to be successfully kindled. In total, thirty-two rats were successful kindled, the success rate was 72.7 %.

### Drug Screening

Then epileptic rats were intraperitoneally injected with PHT (62.5 mg/kg body weight), and were screened for drug resistance. According to the after discharge threshold (ADT), the rats with 100 % increase of ADT after drug administration were considered drug sensitive epileptic rats. Whereas, refractory epileptic rats were ADT did not increase or only increased up to 20 % after drug administration.

### Establishing a Microdialysis System

Microdialysis was established based on previous studies [11]. The animals were implanted with microdialysis cannula into the hippocampus. The cannula was fixed to the skull with dental acrylic cement. The coordinates were 2.0 mm lateral and 3.0 mm posterior to bregma and 3.5 mm ventral starting from the dura according to the animal anatomy atlas. After connecting the microdialysis device, animals received continuous unilateral intrahippocampal perfusion of artificial cerebrospinal fluid at a constant flow rate (2.0 μl/min), then balanced for 1 h to make the substance exchange tends of cerebrospinal fluid within the probe and the hippocampus extracellular fluid to be stable. SB202190 (4 mmol/l) was injected through the microdialysis probe (2.0 μl/min) for half an hour before administrating AEDs in SB202190 group. Finally, 20 μl of exchanged effluent dialysate were collected at various time points (0, 30, 60, 90, 120 and 150 min) after intraperitoneal injection of 200 mg/kg VPA and 10 mg/kg LTG. Microdialysate samples were stored at −80 °C and used for HPLC detection in 2 weeks. Because the permeability of semi permeable membrane was different for each drug, the actual concentration of AEDs in hippocampal extracellular fluid was equal to the concentration of AEDs in dialysate/recovery efficiency of microdialysis probe in vitro.

### Measuring Drug Concentration Using Dialysate by High-Performance Liquid Chromatography

Concentrations of VPA and LTG in dialysate were detected using HPLC. A 20 μl sample was directly injected into the chromatograph (MODEL 510). Chromatographic conditions were as follows: Ultimate XB-C8 column (150 mm × 4.6 mm, 5 μm particle size, for detecting VPA), Dikma C18 column (150 mm × 4.6 mm, 5 μm particle size, for detecting LTG). Mobile phase, methanol/water (55:45, v:v); flow rate, 1.0 ml per minute; ultraviolet wavelength of detection, 210 nm; column temperature, 30 °C; Detector, L-2400 UVD; Column oven, L-2300; Pump, L-2130.

### Immunofluorescence Staining

Frozen Sects. (30 μm) of rat brain tissue were incubated with anti-MRP1 antibody (1:50 dilution, Abcam plc.) and anti-p38 antibody (1:50 dilution, Cell Signaling Technology Inc.) at 4 °C overnight. Then the sections were incubated with DyLight™488-conjugated affinipure secondary antibody (1:500 dilution, Jackson Immuno Research laboratories, Inc.) and DyLight™594-conjugated affinipure secondary antibody (1:300 dilution, Jackson Immuno Research laboratories, Inc.) at 37 °C for an hour. After that, the sections were incubated with DAPI (1:10,000 dilution, Sigma-Aldrich Co.) at 37 °C for 20 min. After sections were mounted and sealed, the results were observed using a fluorescence microscope.

### Western Blotting

Proteins isolated from the hippocampus and cerebral cortex tissue were resolved by SDS-PAGE and transferred to polyvinylidene fluoride (PVDF) membranes. The membranes were incubated with primary antibodies (anti-MRP1 antibody 1:500, dilution, Abcam plc.; anti-p38 MAPK antibody, 1:1000 dilution, Cell Signaling Technology Inc.; anti-p-p38 antibody, 1:1000, dilution, Santa Cruz Biotechnology Inc.; and anti-GAPDH antibody, 1:30,000 dilution, Santa Cruz Biotechnology Inc.) at 4 °C overnight and subsequently horseradish peroxidase-conjugated secondary antibodies at room temperature for 2 h. Specific proteins were visualized with enhanced chemiluminescence detection reagent and determined by an Image Quant LAS4000 mini chemiluminescence imaging system with QUANTITY ONE software (Bio-Rad Laboratories, Hercules, CA, USA).

### Real Time-PCR

Total RNA was extracted from fresh brain tissue using the TRIzol^®^ method. RNA samples were reverse-transcribed to generate first-strand complementary DNA (cDNA). cDNAs were prepared by first strand cDNA synthesis kit according to the protocol. The primers for MRP1, p38, and β-actin were designed by BioTNT Inc. (Shanghai, China). The sequences of gene-specific primers were: MRP1 (ABCC1)-forward: 5′-CGAATGTCCTCTGAGATGGAGAC-3′, reverse: 5′-CTCTACACGGCCTGAATGGG-3′ [12]. β-actin-forward: 5′-GAGCTACGAGCTGCCTGACG-3′, reverse: 5′-GTAGTTTCGTGGATGCCACAG-3′. p38 (MAPK14)- forward: 5′-CGAGCGATACCAGAACCTGT-3′, reverse: 5′-GCGTGAATGATGGACTGAAA-3′. The cycling parameters were as follows: initial denaturation at 95 °C for 10 min, followed by 40 cycles of denaturation 95 °C for 30 s, annealing at 58 °C for 1 min, extension at 72 °C for 1 min. A standard curve and a melting curve were created automatically when the reactions were completed. The mRNA levels of the target genes were quantified with SYBR Green-based real time-PCR analysis (Bio-Rad) and analyzed using the delta delta CT method (ΔΔC_T_) as described and the target amount = $2^{{ - \Delta \Delta C_{\text{T}} }}$ [13].

### Statistical Analysis

Data analysis was made using SPSS11.5. The data are presented as the mean ± standard error. The comparison between samples was examined using the paired *t* test or a one-way ANOVA. Significance was assumed for *p* values <0.05.

## Results

### Down-Regulation of MRP1 Expression in Cortex and Hippocampus of Epileptic Rats

**Fig. 1:**
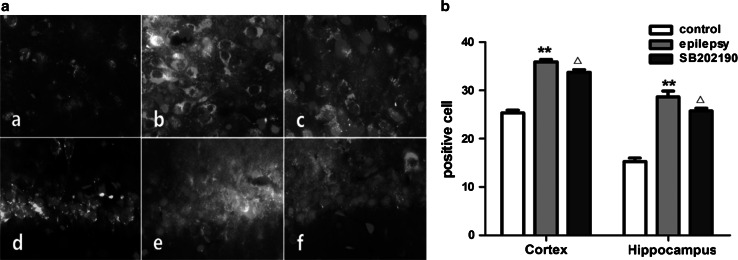
**a** MRP1 and p38 MAPK expression in the rat brain (fluorescence microscope 400×). *a* Cortex of the control group; *b* Cortex of the epilepsy group; *c* Cortex of the SB202190 group; *d* Hippocampus CA1 region of the control group; *e* Hippocampus CA1 region of the epilepsy group; *f* Hippocampus CA1 region of the SB202190 group. **b** Quantitative analysis of immunofluorescence. MRP1-positive and p38 MAPK-positive cells were counted and at least 3 slices from each brain were measured. **p < 0.01 compared with the control group; ^△^ *p* < 0.05 compared with the epilepsy group

Immunofluorescence staining showed that the expression of MRP1 could be observed in the hippocampus and cerebral cortex of rats in each group. The Immunofluorescence images labeled with p38 MAPK (red) exhibiting cytoplasmic location in the control group and nuclear location in the epilepsy and SB202190 groups, while the membrane location for MRP1 (green) and the nuclear location for Dapi (blue) were observed in each group. The MRP1 and p38 MAPK-positive cells were significantly higher in both cortex and hippocampus CA1 region in epilepsy group than those in control group (*p* < 0.01). Compared with the epilepsy group, the number of MRP1 and p38 MAPK-positive cells were significantly decreased in SB202190 group (*p* < 0.05; Fig. 1a, b).

**Fig. 2:**
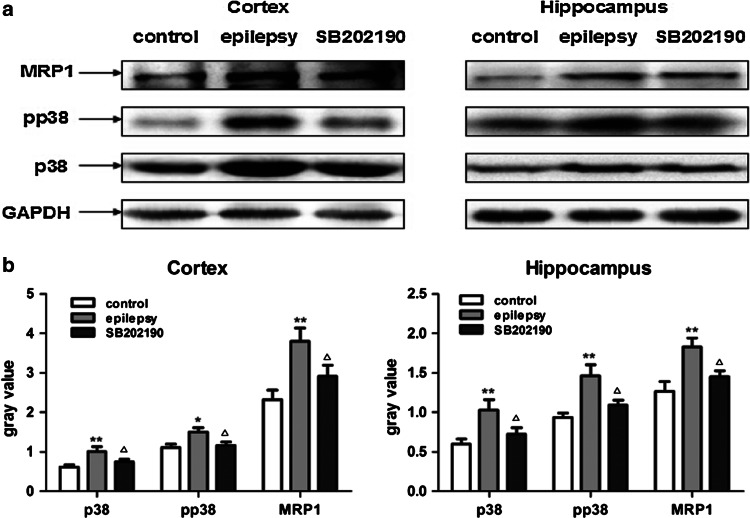
**a** MRP1 and p38 MAPK protein expression in the rat brain. **b** Quantitative analysis of western blot. **p < 0.01, *p < 0.05 compared with the control group; ^△^ *p* < 0.05 compared with the epilepsy group

Similarly, Western blot results showed that the expression of MRP1, p38 and p-p38 protein was significantly increased in the hippocampus and cortex in epilepsy group compared with the control group (*p* < 0.01). After SB202190 administration, the protein expression of MRP1, p38 and p-p38 decreased significantly compared with that in the epilepsy group (*p* < 0.05; Fig. 2a, b).

**Fig. 3:**
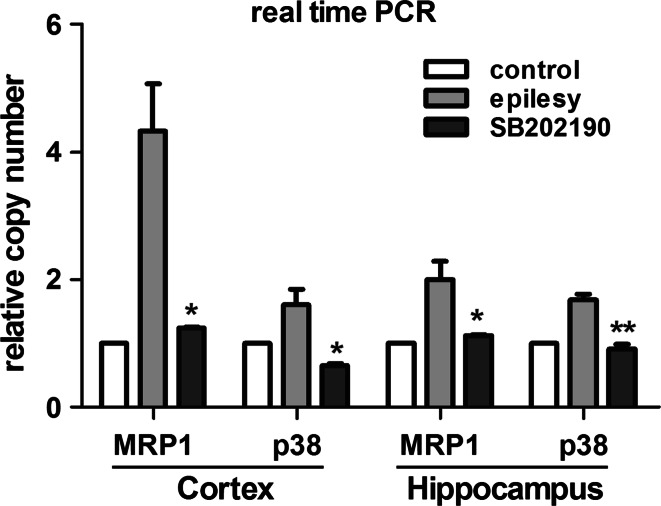
Quantitative analysis of real-time PCR. The target mRNA expression of the control group was recruited as 1. **p < 0.01, *p < 0.05 compared with the epilepsy group

Finally, quantitative real time-PCR (qPCR) analysis revealed that the mRNA expression of MRP1 was significantly higher in the hippocampus and cortex in epilepsy group than that in control group (ratio_hippocampus_ = 2.0070 ± 0.28301, ratio_cortex_ = 4.3231 ± 0.74318), and the same as the mRNA expression of p38 (ratio_hippocampus_ = 1.6947 ± 0.08200, ratio_cortex_ = 1.6155 ± 0.23264). For the SB202190 group, the mRNA expression of MRP1 and p38 was lower than that in the epilepsy group (*p* < 0.05; Fig. 3).

### SB202190 Decreased Seizures Level and Frequency of Epileptic Rat

Through the analysis of the behavior in rats of refractory epilepsy, we found that before the treatment of SB202190, seizures levels were all above stage III, which contained 10 times stage III seizures, 17 times stage IV seizures and 18 times stage V seizures. While after the administration of SB202190, most seizures were at stage IV, with one time stage II seizure, 12 times stage V seizures and 11 times stage III seizures. It showed that seizures level and frequency in the SB202190 group were decreased than that in the epilepsy group (*p* < 0.05; Table 1).

**Table 1:** The effect of SB202190 on the behavior in epilepsy rats

Grading according to the standards of Racine	Behavior before the injection of SB202190 (times)	Behavior after the injection of SB202190 (times)*
I	0	0
II	0	1
III	10	11
IV	17	20
V	18	12

* *p* < 0.05 compared with the epilepsy group

### SB202190 Increased VPA Concentration in the Hippocampal Extracellular Fluid of Epileptic Rat

**Fig. 4:**
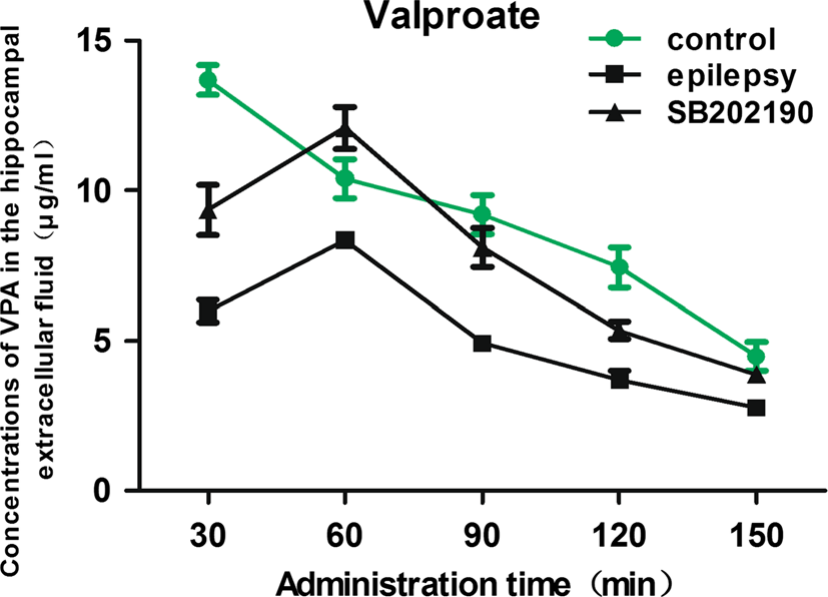
Concentration of VPA in the hippocampal extracellular fluid

VPA was detected in the hippocampal extracellular fluid 30 min after administration in each group. In the control group, the highest concentrations of VPA in the hippocampal extracellular fluid was 13.69 ± 0.48 μg/ml at 30 min post-injection (Fig. 4). Both the epilepsy and the SB202190 group, VPA reached a peak at 60 min and gradually decreased thereafter. However, the concentrations of VPA in the hippocampal extracellular fluid in the epilepsy group were significantly lower at each time point (*p* = 0.000, 0.023, 0.000, 0.000 and 0.002, respectively) compared with those in the control group. In addition, SB202190, a specific p38 MAPK inhibitor, significantly increased VPA concentration in the hippocampal extracellular fluid at each time point post-injection in the SB202190 group (*p* = 0.001, 0.000, 0.001, 0.035 and 0.039 vs. the epilepsy group, respectively). The result indicated that the administration of SB202190 did not change the trend of fluctuation of VPA, but significantly increased the concentrations of VPA in the hippocampal extracellular fluid of epileptic rats.

### SB202190 Increased LTG Concentration in the Hippocampal Extracellular Fluid of Epileptic Rat

**Fig. 5:**
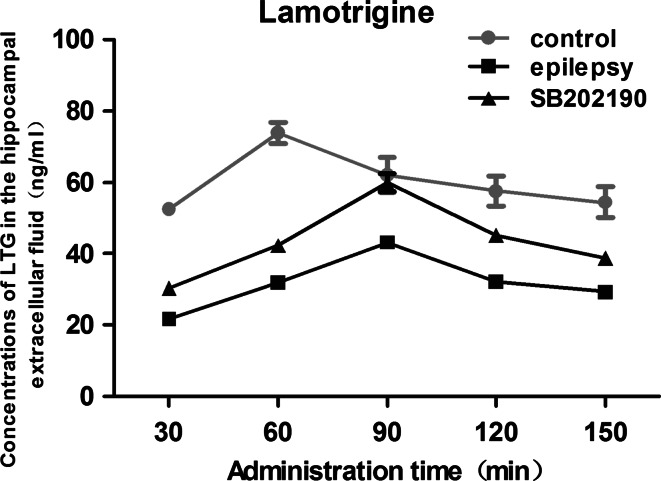
Concentration of LTG in the hippocampal extracellular fluid

Similarly, LTG was detected in the hippocampal extracellular fluid 30 min after administration in each group. In the control group, the highest concentrations of LTG in the hippocampal extracellular fluid was 73.87 ± 2.98 ng/ml at 60 min post-injection (Fig. 5). Both the epilepsy and the SB202190 group, LTG reached a peak at 90 min and gradually decreased thereafter. The LTG level found in the hippocampal extracellular fluid in the epilepsy group was much lower than those in the control group at each time point over 30–150 min (*p* = 0.000, 0.000, 0.002, 0.000 and 0.006, respectively). Moreover, SB202190 administration significantly increased LTG concentrations in the hippocampal extracellular fluid at each time point in the SB202190 group (*p* = 0.007, 0.006, 0.007, 0.010 and 0.046 vs. epilepsy group, respectively). The result indicated that the administration of SB202190 did not change the trend of fluctuation of LTG, but significantly increased the concentrations of LTG in the hippocampal extracellular fluid of epileptic rats.

## Discussion

Almost 30 % of patients with epilepsy are resistant to AEDs therapy [14]. The mechanism of drug resistance is unclear, and likely to be multifactorial, but may include alterations of pharmacological targets and poor penetration of AEDs into the brain because of increased expression of multiple drug-resistance proteins, such as P-glycoprotein and MRP1. MRP1, also known as ABCC1, belongs to the ATP-binding cassette superfamily, which is an important multidrug transporter in the brain [15–17]. It is encoded by a gene located in chromosome 16p13.1 [18] and involved in chemotherapy drug resistance because of its three membrane-spanning domains and two nucleotide-binding domains [19, 20]. The outwardly directed active efflux mechanism, either by extracellular drug export or by intracellular vesicular sequestration, appears to act as an active defense mechanism for limiting brain accumulation of many lipophilic drugs [19, 21, 22]. There is accumulating evidences that overexpression of MRP1 was found in the brain of patients with refractory epilepsy [23, 24]. In addition, several studies have shown that the overexpression of MRP1 causes resistance to antiepileptic drugs [25, 26], due to its function as an efflux pump to transfer AEDs out of the brain tissue or cells [27]. MRP2, another transporter of AEDs in the brain, found mainly in the luminal surface of brain capillary endothelium, whereas other MRPs, such as MRP3 and MRP5, are located basolaterally [28]. It was determined that increased expression of the gene encoding MRP2 by using gene arrays to study mRNAs of multidrug transporters in endothelial cells isolated from surgically resected epileptic foci of patients with pharmacoresistant partial epilepsy [23]. Subsequently, it was shown by other groups that, the concentration of PHT in the brain of MRP2-deficient rats was significantly higher than in the wild-type rats [29], indicating that MRP2 substantially contributes to BBB function.

Most experimental and clinical studies on the multidrug-transporter hypothesis of intractable epilepsy have examined the expression and function of P-gp in the brain, whereas only relatively few studies dealt with other multidrug transporters, such as MRP1 or MRP2. So in our study, we choose MRP1 as the target transporter. In our early studies, we had found that probenecid, MRP inhibitor, significantly increased the concentration of LTG in the extracellular fluid of the hippocampus [30]. For the chosen of AEDs, as we know, the combination of sodium valproate with lamotrigine demonstrating synergism. And VPA belongs to I generation AEDs, while LTG belongs to II generation AEDs. So we choose these two AEDs.

Mitogen-activated protein kinase (MAPK) is an important signaling pathway, which are activated by different extracellular stimuli or by signal transduction from the cell surface to the nucleus. In addition, the regulated MAPK cascade reaction system is involved in cell proliferation, differentiation, apoptosis, migration, invasion and inflammation [31]. There are 5 main subgroups manipulating by a set of sequential actions: ERK (ERK1/ERK2), c-Jun N (JNK/SAPK), p38 MAPK (p38α, p38β, p38γ and p38δ), ERK3/ERK4 and ERK5 [31, 32]. p38 MAPK, discovered in 1993 [33] is selectively activated by MAPK kinases (MKK3/6) [34] and the mechanism is mediated by dual phosphorylation at the Thr-Gly-Tyr motif.

Some recent data showed that blocking the p38 MAPK pathway could down-regulated P-gp expression and reverse the cellular multidrug resistance [4–6]. And it was shown by other groups that the p38 MAPK and ERK pathway were involved in the resistance of cells to mercury by regulation the expression of MRP1 [7]. The mechanism by which MRP1 is overexpressed in the epileptic brain may be similar to PGP, although MRP1 is only 15 % homologous in amino acid sequence to PGP. Their substrates are close and have a considerable overlap [35]. So in the present study, we chose MRP1 as the target protein and applied SB202190, a p38 MAPK specific antagonist [36] as in our previous study [5].

Our data of this study showed that the expression of MRP1 and p38 MAPK in brain of refractory epileptic rats were significantly increased compared with the control group, which could be down-regulated with SB202190. Compared with the control, the concentration of VPA and LTG in hippocampal extracellular fluid in the epilepsy group were lower during 30–150 min after i.p., and, the concentration of VPA and LTG in hippocampal extracellular fluid in the SB202190 group were higher during 30–150 min after i.p. compared with the epilepsy group. In addition, seizures level and frequency in the SB202190 group were decreased than that in the epilepsy group. So in sum, it has demonstrated that SB202190 significantly decreased MRP1 expression and up-regulated the VPA and LTG concentrations in the hippocampal extracellular fluid of refractory epileptic rats.

All above findings reveal that p38 MAPK might be involved in drug resistance of refractory epilepsy by regulation of MRP1 expression. Further research will be helpful for understanding the mechanism of drug resistance in refractory epilepsy and provide novel therapeutic approaches to refractory epilepsy.
